# Complexity of left ventricular strains in response to elevated volumes in healthy adults – Detailed analysis from the three-dimensional speckle-tracking echocardiographic MAGYAR-Healthy Study

**DOI:** 10.1016/j.ijcha.2023.101236

**Published:** 2023-07-06

**Authors:** Attila Nemes, Árpád Kormányos, Zoltán Ruzsa, Alexandru Achim, Nóra Ambrus, Csaba Lengyel

**Affiliations:** Department of Medicine, Albert Szent-Györgyi Medical School, University of Szeged, Szeged, Hungary

**Keywords:** Left ventricular, Volume, Strain, Three-dimensional, Echocardiography

## Abstract

**Introduction:**

Cardiac haemodynamics is described by the Frank-Starling law, which states that the strength of the left ventricular (LV) systolic contraction is related to the LV diastolic filling, with other words LV stroke volume increases as LV volume increases due to the stretching of the myocyte. The purpose of the present study was to examine how the increasing LV volumes affect LV contractility represented by three-dimensional (3D) speckle-tracking echocardiography (3DSTE) -derived LV strains in healthy adults.

**Methods:**

This is post-hoc analysis of the MAGYAR-Healthy Study employing a novel method for technical analysis of echocardiographic datasets. The present study consisted of 301 healthy adults. Due to inferior image quality, 127 subjects have been excluded, therefore the remaining population included 174 subjects (mean age: 32.9 ± 12.1 years, 80 males). All cases have undergone complete two-dimensional Doppler echocardiography extended with 3DSTE.

**Results:**

LV global longitudinal (gLS) and area (gAS) strains were lowest in case of the highest LV end-diastolic volume (EDV). LV global radial (gRS) and 3D (g3DS) strains tendentiously increased with increasing LV-EDV. When segmental analysis was performed, increased LV-EDV was associated with increase of basal LV-RS and LV-3DS. Increased LV strains were associated with increased LV ejection fraction (EF) due to higher LV-EDV for LV-gRS (and LV-g3DS), lower LV-ESV for LV-gCS and lower LV-EDV and LV-ESV for LV-gLS (and LV-gAS). With increasing LV-gRS, LV-gCS and LV-g3DS, all LV strains increased except LV-gLS. With increasing LV-gLS, LV-gRS did not show any increase, LV-gCS and LV-g3DS were the highest when LV-gLS was the highest, while LV-gAS increased simultaneously. With increasing LV-gAS, all LV strains increased.

**Conclusions:**

There is a complex contractility pattern of LV segments/regions in response to elevated LV volumes in healthy circumstances.

## 1 Introduction

Cardiac haemodynamics is described by the Frank-Starling law, which states that the strength of the left ventricular (LV) systolic contraction is related to the LV diastolic filling, with other words, LV stroke volume increases as LV volume increases due to the stretching of the myocyte [1]. The simpliest way to assess LV volumes and contractility at the same time in clinical circumstances is echocardiography due to its non-invasivity and easy-to-perform nature. However, old-fashioned techniques suffer in several technical issues including inaccuracy and calculated results show large deviation. Newly developed methods raise possibility of eliminating the problems detected with older ones. Three-dimensional speckle-tracking echocardiography (3DSTE) seems to be an ideal imaging technique for physiologic studies due to its ability for simultaneous assessment LV volumes and strains, quantitative features of LV contractility, at the same time using the same 3D acquired echocardiographic datasets [2], [3], [4], [5], [6], [7], [8], [9]. Besides in non-invasive nature, 3DSTE does not require significantly more time for a much more detailed analysis in a routine patient. The purpose of the present study was to examine how the increasing LV volumes affect LV contractility represented by 3DSTE-derived LV strains in normal circumstances in healthy.

## 2 Subjects and methods

### 2.1 Subjects

The present study consisted of 301 healthy adults, who were recruited for screening between 2011 and 2015 on a voluntary basis. Due to inferior image quality, 127 subjects have been excluded, therefore the remaining population included 174 subjects (mean age: 32.9 ± 12.1 years, 80 males). All cases have undergone a physical examination, laboratory assessments, standard 12-lead electrocardiography (ECG) and two-dimensional Doppler echocardiography (2DE) with a negative result. No one was taking any medication regularly or had a known disease, pathological state or clinical condition, which could affect the findings. Together with 2DE, 3DSTE-derived data acquisition was also performed at the same time in accordance with our local practices [10], [11]. Detailed analysis was performed later offline using a specific vendor-derived platform. The present study is a part of the **‘M**otion **A**nalysis of the heart and **G**reat vessels b**Y** three-dimension**A**l speckle-t**R**acking echocardiography in **Healthy** subjects’ **(MAGYAR-Healthy) Study,** which has been organized at the University of Szeged partly for physiologic analyses evaluating interplay between volumetric and functional properties of cardiac chambers (’Magyar’ means’Hungarian’ in Hungarian language). The study was conducted in accordance with the Declaration of Helsinki (as revised in 2013) and was approved by the Institutional and Regional Human Biomedical Research Committee of University of Szeged, Hungary (No.: 71/2011 and updated versions) and informed consent was given by all subjects.

### 2.2 Two-dimensional Doppler echocardiography

For complete echocardiographic analysis, all healthy subjects have undergone 2DE. Cardiac chamber quantifications were performed according to recent guidelines including measurement of LV dimensions and volumes, determination of LV ejection fraction by the Simpson’s method, Doppler exclusion of valvular regurgitations or stenoses, and Doppler measurement of diastolic mitral inflow velocities and their ratio (E/A) [12]. Toshiba Artida™ echocardiographic tool (Toshiba Medical Systems, Tokyo, Japan) with a PST-30BT (1–5 MHz) phased-array transducer was used in all cases.

### 2.3 Three-dimensional speckle-tracking echocardiography

Detailed LV analysis was performed in all cases including determination of LV volumes and strains, as well [7], [8]. Firstly, 3DSTE-derived 3D echocardiographic datasets were acquired using the same Toshiba Artida™ echocardiographic machine (Toshiba Medical Systems, Tokyo, Japan), but transducer was changed to a PST-25SX matrix transducer with 3D capability. All subjects were lying in left lateral decubitus position, transducer was positioned apically, cases were in sinus rhythm, breath was asked to be hold during data acquisitions. For optimal image quality, 6 subvolumes were acquired within 6 cardiac cycles, software merged them together automatically for a full volume 3D echocardiographic dataset.

At second stage, offline data analysis was performed with a vendor-provided 3D Wall Motion Tracking software version 2.7 (Ultra Extend, Toshiba Medical Systems, Tokyo, Japan). Optimal apical longitudinal views were selected on apical 4-chamber (AP4CH) and 2-chamber (AP2CH) views and cross-sectional views at basal, midventricular and apical levels. For detailed LV analysis, septal mitral annular (MA)-LV edge, endocardial LV apical surface and lateral MA-LV edge were determined, then a sequential analysis was started creating a virtual 3D model of the LV. With this LV cast, LV volume changes could be determined during the cardiac cycle together with exact detection of LV-EF and objective features of LV contractility called as LV strains. Global (evaluating the whole LV), segmental (only for a certain segment), mean segmental (evaluating from 16-segments of the LV as a mean value) and regional (for basal, midventricular and apical regions of the LV from segmental values) strains were calculated [2], [3], [4], [5].

**Fig. 1:**
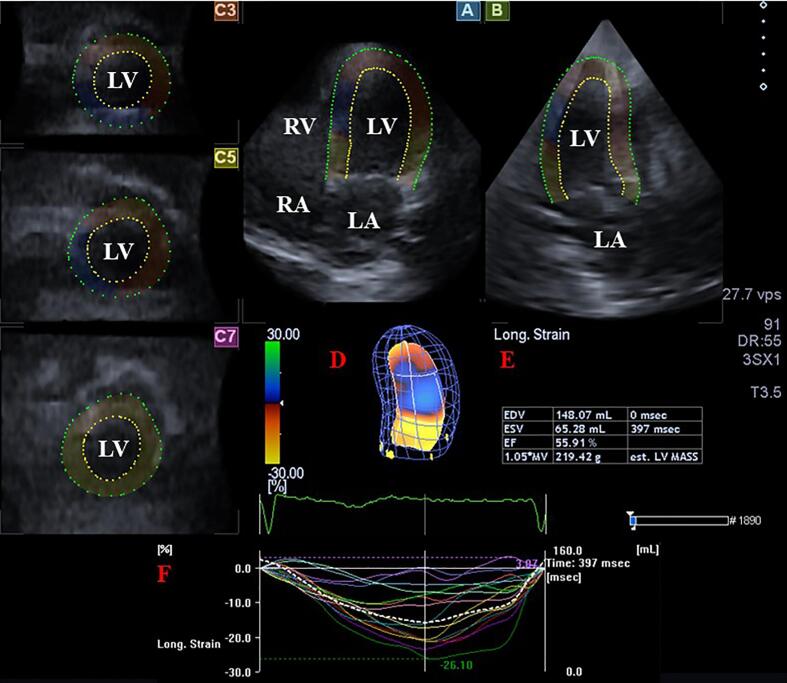
Three-dimensional (3D) speckle-tracking echocardiographic evaluation of the left ventricle (LV). Apical longitudinal four-chamber (A) and two-chamber views (B) and apical (C3), midventricular (C5) and basal (C7) short-axis views are seen together with 3D cast of the LV (red D), volumetric LV parameters and LV ejection fraction (red E), time-LV volume changes (dashed white line) and global (white line) and segmental (coloured lines) time-LV longitudinal strain curves. **Abbreviations.** LV = left ventricle, LA = left atrium, RA = right atrium, RV = right ventricle. (For interpretation of the references to colour in this figure legend, the reader is referred to the web version of this article.)

Strains were measured for featuring thickening and thinning of the myocardium (radial strain, RS), lengthening and shortening of the myocardium (longitudinal strain, LS) and widening and narrowing of the myocardium (circumferential strain, CS). Based on these unidirectional/unidimensional strains, complex/multidimensional strains were also determined: area strain (AS) as a combination of LS and CS and 3D strain (3DS) as a combination of RS, LS and CS [2], [3], [4], [5] (see Fig. 1).

### 2.4 Statistical analysis

Continuous variables are presented in mean ± standard deviation format, while categorical variables are presented in number/percentage format. The p < 0.05 was considered to be significant. Fischer’s exact test was used for all categorical variables. To test normality of distribution for continuous variables, Shapiro-Wilks test was performed: in case of normal distribution Student’s *t*-test was used, in case of non-normal distribution, Mann-Whitney-Wilcoxon test was performed. One-way analysis of variance (ANOVA) test with Bonferroni correction were used, where appropriated. For intraobserver and interobserver correlations, intraclass correlation coefficients (ICCs) were calculated. Statistical analyses were performed with SPSS software version 22 (SPSS Inc., Chicago, IL, USA).

## 3 Results

### 3.1 Clinical data

All subject involved proved to be non-smoker with a family history of cardiovascular disorders in 40 out of 174 cases (23%). Systolic (123.1 ± 5.3 mmHg) and diastolic (76.1 ± 5.1 mmHg) blood pressures and heart rate (72.3 ± 2.4 1/s) were in normal ranges. Mean height, weight and calculated body surface area proved to be 171.1 ± 8.1 cm, 72.5 ± 11.2 kg and 1.86 ± 0.21 kg/m^2^.

### 3.2 Two-dimensional Doppler echocardiographic analysis

Routine 2D echocardiographic data including left atrial diameter measured in parasternal long-axis view (36.6 ± 4.0 mm), LV end-diastolic diameter (48.1 ± 3.7 mm) and volume (106.8 ± 22.8 ml), LV end-systolic diameter (39.9 ± 25.0 mm) and volume (36.5 ± 9.2 ml), interventricular septum (9.0 ± 1.5 mm), LV posterior wall (9.1 ± 1.6 mm) and LV ejection fraction (65.9 ± 4.9%) were in normal range. Mean diastolic mitral inflow velocities (E and A) were 80.2 ± 17.5 cm/s and 64.6 ± 19.7 cm/s, respectively. No subject showed larger than grade 1 valvular regurgitation or had valvular stenosis on any valves.

### 3.3 Classification of subjects

The mean ± SD of LV-EDV, LV-ESV, LV-gRS, LV-gCS, LV-gLS, LV-gAS and LV-g3DS of adult healthy subjects proved to be 86.1 ± 23.2 ml, 36.2 ± 10.6 ml, 25.4 ± 9.4%, −27.7 ± 5.0%, −16.1 ± 2.5%, −40.4 ± 4.9% and 28.0 ± 9.0%, respectively. The population of healthy cases were classified into the following subgroups:

– subjects having less than (mean – 1SD) of the above mentioned parameters (62.9 ml, 25.6 ml, 18.8%, 22.7%, 13.6%, 35.5% and 19.0%, respectively),
– subjects having larger than (mean + SD) of the above mentioned parameters (109.3 ml, 46.8 ml, 36.6%, 32.7%, 18.6%, 45.3% and 37.0%, respectively),
– subjects having larger/equal than (mean – 1SD) and lower/equal than (mean + 1SD) of the above mentioned parameters.

### 3.4 Three-dimensional speckle-tracking echocardiographic analysis

The mean data acquisition time together with complete 3DSTE-derived LV analysis did not take more than 15 min in any of cases.

Increasing LV-EDV was associated with increasing LV-ESV and LV mass with preservation of LV-EF. Similar happens with increasing LV-ESV as seen in case of LV-EDV with reduction of LV-EF. LV-gLS and LV-gAS were lowest in case of the highest LV-EDV. LV-gRS and LV-g3DS tendentiously increased with increasing LV-EDV. With increasing LV-ESV lower LV-gLS, LV-gCS and LV-gAS could be detected (Table 1).

**Table 1:** Left ventricular volumes and global/mean segmental strains in different left ventricular volume groups.

	**EDV < 62.9 ml** **(n = 22)**	**62.9 ml ≤ EDV ≤ 109.3 ml** **(n = 131)**	**109.3 ml < EDV** **(n = 21)**	**ESV < 25.6 ml** **(n = 23)**	**25.6 ml ≤ ESV ≤ 46.8 ml** **(n = 127)**	**46.8 ml < ESV** **(n = 24)**
**EDV (ml)**	56.3 ± 5.1	84.8 ± 12.2*	131.1 ± 18.7*	62.3 ± 12.1	83.0 ± 15.0*	125.5 ± 21.0*
**ESV (ml)**	23.5 ± 4.8	35.5 ± 6.8*	56.2 ± 8.1*	21.8 ± 3.1	35.2 ± 5.8*	55.2 ± 7.5*
**EF (ml)**	58.8 ± 7.5	58.4 ± 5.4	57.2 ± 4.0	64.6 ± 5.9	57.6 ± 5.0*	55.8 ± 3.9*
**3D mass (g)**	130.2 ± 22.9	157.8 ± 29.1*	188.9 ± 32.7*	130.4 ± 21.7	157.0 ± 29.0*	189.0 ± 30.0*
**g RS (%)**	22.6 ± 9.9	25.6 ± 9.5	27.0 ± 7.4	26.9 ± 8.2	24.8 ± 10.0	27.1 ± 6.7
**g CS (%)**	−28.6 ± 6.8	−27.7 ± 4.9	−26.8 ± 3.2	–32.9 ± 5.4	−24.8 ± 4.9§	−26.2 ± 3.2§
**g LS (%)**	16.8 ± 2.7	−16.2 ± 2.3	−14.6 ± 2.9*†	−17.2 ± 2.4	−16.1 ± 2.3§	−14.9 ± 2.8§‡
**g AS (%)**	−41.9 ± 5.8	−40.4 ± 4.8	−38.4 ± 4.1*	−45.6 ± 4.9	−39.8 ± 4.5§	−38.1 ± 3.8§
**g 3DS (%)**	25.2 ± 10.4	28.1 ± 9.0	30.3 ± 6.8	30.2 ± 9.1	27.2 ± 9.4	30.1 ± 6.4
**ms RS (%)**	25.6 ± 9.5	27.8 ± 9.1	29.2 ± 6.5	29.3 ± 8.0	27.1 ± 9.4	29.1 ± 6.1
**ms CS (%)**	−29.6 ± 6.7	−28.6 ± 4.7	−27.6 ± 2.9	–33.6 ± 5.3	−29.2 ± 5.5§	−27.1 ± 3.2§
**ms LS (%)**	−17.7 ± 3.0	−17.0 ± 2.2	−15.5 ± 2.6*†	−18.1 ± 2.7	−16.9 ± 2.2§	−15.7 ± 2.6§‡
**ms AS (%)**	−42.7 ± 5.7	−41.4 ± 4.7	−38.8 ± 4.5*†	−46.5 ± 4.7	−40.8 ± 4.4§	−38.5 ± 3.9§‡
**ms 3DS (%)**	27.6 ± 10.0	30.2 ± 8.8	34.3 ± 4.7*	32.6 ± 8.6	29.2 ± 9.2	32.9 ± 5.2

**Abbreviations:** EDV = end-diastolic volume, ESV = end-systolic volume, EF = ejection fraction, 3D = three-dimensional, g = global, ms = mean segmental, RS = radial strain, CS = circumferential strain, LS = longitudinal strain, AS = area strain, 3DS = three-dimensional strain *p < 0.05 vs. EDV < 62.9 ml; †p < 0.05 vs. 62.9 ml ≤ EDV ≤ 109.3 ml; §p < 0.05 vs. ESV < 25.6 ml; ‡p < 0.05 vs. 25.6 ml ≤ ESV ≤ 46.8 ml.

When segmental analysis was performed, increased LV-EDV was associated with increase of basal LV-RS and LV-3DS, decrease of midventricular LV-LS and LV-AS and reduction of apical LV-CS. Increased LV-ESV showed associations with reduced LV-CS in all regions, decrease of midventricular LV-LS and deterioration of LV-AS in all regions (Table 2).

**Table 2:** Left ventricular segmental strains in different left ventricular volume groups.

	**EDV < 62.9 ml** **(n = 22)**	**62.9 ml ≤ EDV ≤ 109.3 ml** **(n = 131)**	**109.3 ml < EDV** **(n = 21)**	**ESV < 25.6 ml** **(n = 23)**	**25.6 ml ≤ ESV ≤ 46.8 ml** **(n = 127)**	**46.8 ml < ESV** **(n = 24)**
**RS_basal_ (%)**	27.6 ± 11.5	31.6 ± 13.4	36.1 ± 9.6*	32.6 ± 10.3	30.7 ± 3.8	35.4 ± 9.5
**RS_mid_ (%)**	28.5 ± 12.6	30.2 ± 10.7	30.1 ± 9.6	31.6 ± 12.0	29.6 ± 10.9	30.3 ± 8.6
**RS_apical_ (%)**	18.2 ± 11.3	18.2 ± 8.5	17.5 ± 8.9	20.9 ± 10.4	17.7 ± 8.8	17.8 ± 7.4
**CS_basal_ (%)**	−24.2 ± 5.7	−25.5 ± 5.2	−26.5 ± 4.1	−28.7 ± 6.3	−24.8 ± 4.9§	−26.0 ± 4.0
**CS_mid_ (%)**	−30.8 ± 8.3	−29.8 ± 5.8	−27.6 ± 3.4	−35.5 ± 7.1	−29.2 ± 5.5§	−27.0 ± 3.6§
**CS_apical_ (%)**	−35.9 ± 11.5	−31.4 ± 9.8	−29.1 ± 10.0*	−38.2 ± 9.2	−31.0 ± 10.0§	−28.8 ± 9.5§
**LS_basal_ (%)**	−21.1 ± 4.4	−19.9 ± 4.6	−18.6 ± 4.4	−20.6 ± 3.9	−19.9 ± 4.8	−19.0 ± 4.3
**LS_mid_ (%)**	−13.9 ± 5.2	−13.8 ± 3.5	−11.4 ± 4.4†	−15.5 ± 4.2	−13.6 ± 3.7§	−11.7 ± 4.3§‡
**LS_apical_ (%)**	−18.0 ± 6.1	−17.3 ± 5.6	−17.4 ± 6.4	−18.2 ± 6.4	−17.3 ± 5.6	−17.0 ± 5.8
**AS_basal_ (%)**	−39.2 ± 5.7	−39.9 ± 5.3	−39.9 ± 5.3	−42.6 ± 6.3	−39.3 ± 5.9§	−39.6 ± 5.2
**AS_mid_ (%)**	−41.6 ± 8.4	−40.4 ± 5.9	−36.3 ± 5.9*†	−46.5 ± 7.2	−39.7 ± 5.5§	−36.0 ± 5.4§‡
**AS_apical_ (%)**	−49.3 ± 11.6	−44.5 ± 11.4	−42.3 ± 12.3	−51.3 ± 10.5	−44.2 ± 11.5§	−42.1 ± 11.5§
**3DS_basal_ (%)**	30.2 ± 11.5	35.1 ± 12.7	40.2 ± 9.2*	37.2 ± 10.6	33.9 ± 13.0	39.1 ± 9.3
**3DS_mid_ (%)**	29.8 ± 12.7	31.6 ± 10.4	32.7 ± 8.5	33.0 ± 12.2	31.0 ± 10.6	32.6 ± 7.8
**3DS_apical_ (%)**	20.4 ± 12.7	19.9 ± 8.9	19.5 ± 6.8	23.5 ± 11.9	19.3 ± 9.2	19.8 ± 7.6

**Abbreviations:** EDV = end-diastolic volume, ESV = end-systolic volume, RS = radial strain, CS = circumferential strain, LS = longitudinal strain, AS = area strain, 3DS = three-dimensional strain, mid = midventricular *p < 0.05 vs. EDV < 62.9 ml; †p < 0.05 vs. 62.9 ml ≤ EDV ≤ 109.3 ml; §p < 0.05 vs. ESV < 25.6 ml; ‡p < 0.05 vs. 25.6 ml ≤ ESV ≤ 46.8 ml.

Increased LV strains were associated with increased LV-EF, but due to higher LV-EDV for LV-gRS (and LV-g3DS), lower LV-ESV for LV-gCS and lower LV-EDV and LV-ESV for LV-gLS (and LV-gAS). Increased LV-gLS and LV-gAS were associated with reduced LV mass (Table 3, Table 4).

**Table 3:** Left ventricular segmental strains in different global left ventricular radial, circumferential and longitudinal strain groups.

	**LV-gRS < 18.8%** **(n = 42)**	**18.8% ≤ LV-gRS ≤ 36.6%** **(n = 111)**	**36.6% < LV-gRS** **(n = 21)**	**LV-gCS < –22.7%** **(n = 20)**	**–22.7% ≤ LV-gCS ≤ –32.7%** **(n = 128)**	**–32.7% < LV-gCS** **(n = 26)**	**LV-gLS < -13.6%** **(n = 25)**	**−13.6% ≤ LV-gLS ≤ -18.6%** **(n = 126)**	**−18.6% < LV-gLS** **(n = 23)**
**EDV (ml)**	79.3 ± 22.8	87.8 ± 24.5*	87.1 ± 20.7	80.0 ± 17.0	87.9 ± 24.9	82.1 ± 16.8	94.1 ± 26.2	86.0 ± 22.0	77.9 ± 24.7#
**ESV (ml)**	35.9 ± 8.7	36.7 ± 11.1	33.6 ± 11.5	37.8 ± 8.9	37.8 ± 10.6	27.0 ± 7.0‡	41.2 ± 13.2	36.1 ± 9.8#	30.7 ± 9.4#&
**EF (ml)**	55.4 ± 4.4	58.7 ± 5.2*	62.1 ± 6.9*†	52.7 ± 4.6	57.3 ± 3.5§	67.4 ± 4.6§‡	56.2 ± 5.4	58.0 ± 5.2	62.5 ± 6.1#&
**3D mass (g)**	151.7 ± 30.1	159.3 ± 32.4	163.6 ± 33.1	157.0 ± 29.6	159.5 ± 32.3	150.3 ± 33.4	171.6 ± 31.3	156.6 ± 32.6#	148.5 ± 26.3#
**g RS (%)**	14.6 ± 3.5	26.2 ± 4.5*	43.2 ± 6.5*†	20.7 ± 8.3	24.4 ± 8.1	34.0 ± 10.8§‡	26.9 ± 12.2	24.6 ± 8.4	28.3 ± 10.6
**g CS (%)**	−25.2 ± 4.0	−28.1 ± 4.8*	−30.6 ± 6.1*†	−20.1 ± 2.4	−27.1 ± 2.7§	−36.2 ± 2.7§‡	−27.0 ± 4.6	−27.3 ± 4.7	−30.7 ± 5.8#&
**g LS (%)**	−15.5 ± 2.2	−16.4 ± 2.4	−16.2 ± 3.4	−15.7 ± 2.2	−16.0 ± 2.3	−16.9 ± 3.2	−12.3 ± 1.5	−16.1 ± 1.4#	−20.0 ± 1.1#&
**g AS (%)**	−37.8 ± 3.7	−41.0 ± 4.7*	−42.7 ± 6.6*	–33.6 ± 3.0	−39.8 ± 3.1§	−48.1 ± 3.6§‡	−37.1 ± 3.8	−40.1 ± 4.4#	−45.6 ± 4.8#&
**g 3DS (%)**	18.0 ± 3.4	28.7 ± 4.8*	45.0 ± 6.2*†	23.1 ± 8.0	26.9 ± 7.7§	37.3 ± 10.0§‡	29.1 ± 11.1	27.2 ± 8.1	31.5 ± 10.8&
**ms RS (%)**	17.9 ± 2.9	28.2 ± 4.4*	44.7 ± 6.9*†	24.0 ± 7.4	26.6 ± 7.7	35.9 ± 10.5§‡	29.2 ± 11.6	26.9 ± 7.8	30.2 ± 10.6
**ms CS (%)**	−26.1 ± 3.8	−29.0 ± 4.5*	−31.6 ± 6.1*†	−21.6 ± 2.6	−27.9 ± 2.6§	−37.0 ± 2.6§‡	−28.0 ± 4.4	−28.2 ± 4.5	−31.5 ± 5.8#&
**ms LS (%)**	−16.3 ± 2.1	−17.1 ± 2.3	−17.0 ± 3.2	−16.5 ± 1.9	−16.8 ± 2.3	−17.7 ± 3.0	−13.5 ± 1.4	−16.9 ± 1.4#	−20.7 ± 1.7#&
**ms AS (%)**	−39.0 ± 3.9	−41.8 ± 4.5*	−44.0 ± 6.7*	−34.5 ± 3.0	−40.6 ± 3.1§	−49.0 ± 3.5§‡	−38.3 ± 4.0	−41.1 ± 4.3#	−46.4 ± 5.0#&
**ms 3DS (%)**	20.2 ± 3.3	30.5 ± 4.5*	46.7 ± 6.7*†	24.6 ± 6.6	29.1 ± 7.5§	38.8 ± 10.4§‡	31.9 ± 10.7	29.2 ± 7.7	33.4 ± 11.2&

**Abbreviations:** EDV = end-diastolic volume, ESV = end-systolic volume, RS = radial strain, CS = circumferential strain, LS = longitudinal strain, AS = area strain, 3DS = three-dimensional strain, mid = midventricular *p < 0.05 vs. LV-gRS < 18.8%, †p < 0.05 vs. 18.8% ≤ LV-gRS ≤ 36.6%, §p < 0.05 vs. LV-gCS < –22.7%, ‡p < 0.05 vs. –22.7% ≤ LV-gCS ≤ –32.7%, #p < 0.05 vs. LV-gLS < −13.6%, &p < 0.05 vs. −13.6% ≤ LV-gLS ≤ −18.6%.

**Table 4:** Left ventricular volumes and global/mean segmental strains in different global left ventricular area and three-dimensional strain groups.

	**LV-gAS < -35.5%** **(n = 24)**	**−35.5% ≤ LV-gAS ≤ -45.3%** **(n = 125)**	**−45.3% < LV-gAS** **(n = 25)**	**LV-g3DS < 19%** **(n = 26)**	**19% ≤ LV-g3DS ≤ 37%** **(n = 123)**	**37% < LV-g3DS** **(n = 25)**
**EDV (ml)**	89.2 ± 22.9	87.2 ± 24.3	78.2 ± 16.0	80.3 ± 20.9	87.1 ± 24.2	87.7 ± 20.1
**ESV (ml)**	41.7 ± 10.1	37.3 ± 10.1*	25.3 ± 5.5*†	36.2 ± 8.4	36.7 ± 10.9	33.7 ± 11.4
**EF (ml)**	52.8 ± 4.4	57.4 ± 3.4	67.7 ± 4.2*†	54.5 ± 4.7	58.3 ± 4.9§	62.2 ± 6.8§‡
**3D mass (g)**	165.5 ± 35.2	159.3 ± 31.6	143.0 ± 28.0*†	157.5 ± 28.8	157.2 ± 32.7	161.5 ± 33.5
**g RS (%)**	22.2 ± 11.0	24.5 ± 7.6	32.6 ± 12.2*†	13.2 ± 3.9	24.6 ± 4.9§	41.7 ± 7.2§‡
**g CS (%)**	−21.2 ± 3.1	−27.2 ± 3.0*	−36.1 ± 2.9*†	−25.1 ± 4.4	−27.6 ± 4.6§	−30.8 ± 6.0§‡
**g LS (%)**	−14.1 ± 2.2	−16.1 ± 2.2*	−18.2 ± 2.5*†	−15.7 ± 2.2	−16.2 ± 2.4	−16.2 ± 3.2
**g AS (%)**	–33.2 ± 2.2	−40.0 ± 2.7*	−48.9 ± 2.8*†	−37.8 ± 3.9	−40.4 ± 4.5§	−42.8 ± 6.4§‡
**g 3DS (%)**	24.8 ± 10.4	27.0 ± 7.2	36.0 ± 11.5*†	16.1 ± 3.0	27.3 ± 4.7§	44.0 ± 6.1§‡
**ms RS (%)**	25.2 ± 9.9	26.8 ± 7.2	34.5 ± 12.1*†	16.8 ± 3.7	26.7 ± 4.7§	43.4 ± 7.1§‡
**ms CS (%)**	–22.6 ± 3.0	−28.1 ± 2.9*	−36.8 ± 2.9*†	−26.1 ± 4.3	−28.5 ± 4.3§	−31.7 ± 6.0§‡
**ms LS (%)**	−14.9 ± 1.9	−16.8 ± 2.0*	−19.0 ± 2.8*†	−16.5 ± 2.1	−16.9 ± 2.3	−17.0 ± 3.0
**ms AS (%)**	−34.2 ± 2.1	−40.8 ± 2.7*	−49.7 ± 2.7*†	−38.5 ± 4.1	−41.5 ± 4.4§	−43.8 ± 6.5§‡
**ms 3DS (%)**	27.5 ± 9.9	29.1 ± 7.0	37.4 ± 12.0*†	18.6 ± 2.8	29.3 ± 4.4§	46.0 ± 6.5§‡

**Abbreviations:** EDV = end-diastolic volume, ESV = end-systolic volume, EF = ejection fraction, 3D = three-dimensional, g = global, ms = mean segmental, RS = radial strain, CS = circumferential strain, LS = longitudinal strain, AS = area strain, 3DS = three-dimensional strain *p < 0.05 vs. LV-gAS < 35.5%, †p < 0.05 vs. −35.5% ≤ LV-gAS ≤ -45.3%, §p < 0.05 vs. LV-g3DS < 19%, ‡p < 0.05 vs. 19% ≤ LV-g3DS ≤ 37%.

With increasing LV-gRS, LV-gCS and LV-g3DS, all LV strains increased except LV-LS. With increasing LV-gLS, LV-gRS did not show any increase, LV-gCS and LV-g3DS were the highest when LV-gLS was the highest, while LV-gAS increased simultaneously. With increasing LV-gAS, all LV strain increased (Table 3, Table 4).

### 3.5 Feasibility of 3DSTE-derived parameters

All measurements were peformed 5 times in all cases. Adequate simultaneous LV volumetric and strain measurements could be performed in 174 out of 301 assessments (58% overall feasibility).

### 3.6 Reproducibility of 3DSTE-derived parameters

Intraobserver ICCs for LV-EDV, LV-ESV, LV-RS, LV-CS, LV-LS, LV-3DS and LV-AS proved to be 0.91, 0.90, 0.85, 0.81, 0.82, 0.80, and 0.83, respectively. Interobserver ICCs for the same parameters were 0.92, 0.91, 0.82, 0.78, 0.79, 0.78, and 0.80, respectively.

## 4 Discussion

Strain and strain rate are measures of deformation featuring left ventricular (LV) function. Deformation represented by strain reflects the change in dimensions of myocardial fibres over the cardiac cycle, strain rate is the change in strain with respect to time [13]. Given that a certain LV wall segment/region is capable of deforming in 3 directions of space, unidimensional/unidirectional LV-gRS, LV-gLS and LV-gCS are used to assess LV deformation. Although the heart is the driver of haemodynamic system, circulation is highly dependent on homeostatic mechanisms of autoregulation, quality of blood, vascular resistance and tension, blood pressure, and several other factors [14]. It is known, that LV systolic contraction is strongly related to LV diastolic volume as described by the Frank-Starling law: as the LV diastolic volume is higher, the LV-SV is more increased [1]. Based on all these facts, it is reasonable to ask what changes can be observed in the LV strain pattern in case of increasing LV volume and what LV volumes can be observed in case of elevated LV strains.

2DSTE is a widely used echocardiographic technique for LV-gLS assessment with a significant prognostic impact [15]. In addition to LV-LS, LV-RS and LV-CS can also be determined following changing of evaluation planes, which makes the whole analysis time-consuming and complicated. Theoretic benefit of 2DSTE-derived strain assessments is its simplicity, however, the disadvantage is that examines LV only in a specific plane [15].

3DSTE is a novel imaging method capable of simultaneous assessment of LV volumes (and LV-EF) and strains from the same acquired 3D echocardiographic dataset allowing the examination of their dependence on each other [2], [3], [4], [5]. 3DSTE is validated for volumetric and strain measurements [6], [7], [8], [9], their normal reference values are also published [10], [11]. However, it should be noted that 3DSTE-derived LV-EF is lower compared to what is calculated by 2D echocardiography due to underestimation of LV volumes, in which LV-EDV in more affected [10]. The main benefit of 3DSTE is its ability to assess LV myocardial contraction and its quantitative features in the 3D space: each muscle area (segment, region) contracts and moves in radial, longitudinal and circumferential directions at the same time, which can be quantified by LV-RS, LV-LS and LV-CS unidimensional/unidirectional strains [2], [3], [4], [5]. Due to the special architecture of subendocardial and subepicardial oblique muscle fibres/bands perpendicular to each other, combined strains (LV-AS and LV-3DS) were preferred as approximate characteristics of the physiological state [2], [3], [4], [5]. In normal circumstances, LV-RS (and LV-3DS) with a positive sign increases in systole representing myocardial wall thickening and being largest at end-systole, while LV-LS and LV-CS represent same-time LV shortening and narrowing with a negative sign. This sort of evaluation could help us understand how LV reacts during the cardiac cycle to increased LV volumes.

The main finding of the present study is that increased LV-g3DS, which represents the sum of contractions in all directions, could be detected in healthy subjects with elevated LV-EDV. Segmental analysis showed increase of LV-RS and LV-3DS of basal segments, which seems logical due to its adjacent localization to LV inflow mitral annulus, so this area is affected/stretched firstly by LV volume in diastole. Elevated LV-EDV is accompanied with non-significant tendentious increase of LV-gRS, representing radial contraction of the myocardium, and significant reduction of LV-gLS representing longitudinal myocardial contraction, mainly located in the midventricular region. It should be noted that LV-EF remains preserved. With increasing global LV strains, LV-EF showed simultaneous increase due to elevation of LV-EDV for LV-gRS, reduction of LV-ESV for LV-gCS and reduction of both LV volumes but in different degrees for LV-gLS. With increasing LV-gRS and LV-gCS, all other strains increased simultaneously except LV-gLS, which remained unchanged.

These results suggest a complex contractility pattern of LV segments/regions in response to higher LV volumes. It seems to be important which myocardial areas (like basal regions) are stretched firstly in response to elevated LV volumes making these areas responsible for elevated contractions. Longitudinal contraction, however, seems to compensate for these movements making LV-EF constant. However, further clinical and experimental studies are warranted to confirm our findings, even in different pathological states with higher LV volumes.

### 4.1 Limitation section

Although significant improvement in 3D echocardiographic technology has been performed in the last decade, image quality for 2DE is still better than that of 3DSTE due to limited temporal and spatial resolution. During 3DSTE analysis, 4–6 cardiac cycles and ECG-gated capture are required for optimal virtual 3D images, which make an opportunity for stitching artifacts. Moreover, respiratory motion and rhythm disturbances make data acquisition and imaging difficult or impossible. This sort of limitations could significantly affect the findings [2], [3], [4], [5]. 3DSTE has been demonstrated to have the ability of chamber quantification of atria as well at the same time. However, the present study did not purpose to make such assessments [2], [3], [4], [5], [16], [17]. Moreover, LV rotational parameters were also not aimed to be measured. Although there can be a debate, whether ventricular septum is a part of which ventricle, it was considered to be a part of the LV in this study. The present study did not consider validation of LV volumes and strains [6], [7], [8], [9], and determination of normal references of these parameters due to their validated and determined nature [6], [7], [8], [9], [10], [11].

## 5 Conclusions

There is a complex contractility pattern of LV segments/regions in response to elevated LV volumes in healthy circumstances.

### CRediT authorship contribution statement

**Attila Nemes:** Conceptualization, Writing – original draft, Writing – review & editing. **Árpád Kormányos:** Methodology, Investigation, Data curation. **Zoltán Ruzsa:** Writing – review & editing. **Alexandru Achim:** Writing – review & editing. **Nóra Ambrus:** Writing – review & editing. **Csaba Lengyel:** Writing – review & editing.

## Declaration of Competing Interest

The authors declare that they have no known competing financial interests or personal relationships that could have appeared to influence the work reported in this paper.
